# Accelerating Computation of DCM for ERP in MATLAB by External Function Calls to the GPU

**DOI:** 10.1371/journal.pone.0066599

**Published:** 2013-06-26

**Authors:** Wei-Jen Wang, I-Fan Hsieh, Chun-Chuan Chen

**Affiliations:** 1 Department of Computer Science and Information Engineering, National Central University, Taoyuan, Taiwan; 2 Graduate Institute of Biomedical Engineering, National Central University, Taoyuan, Taiwan; McGill University, Canada

## Abstract

This study aims to improve the performance of Dynamic Causal Modelling for Event Related Potentials (DCM for ERP) in MATLAB by using external function calls to a graphics processing unit (GPU). DCM for ERP is an advanced method for studying neuronal effective connectivity. DCM utilizes an iterative procedure, the expectation maximization (EM) algorithm, to find the optimal parameters given a set of observations and the underlying probability model. As the EM algorithm is computationally demanding and the analysis faces possible combinatorial explosion of models to be tested, we propose a parallel computing scheme using the GPU to achieve a fast estimation of DCM for ERP. The computation of DCM for ERP is dynamically partitioned and distributed to threads for parallel processing, according to the DCM model complexity and the hardware constraints. The performance efficiency of this hardware-dependent thread arrangement strategy was evaluated using the synthetic data. The experimental data were used to validate the accuracy of the proposed computing scheme and quantify the time saving in practice. The simulation results show that the proposed scheme can accelerate the computation by a factor of 155 for the parallel part. For experimental data, the speedup factor is about 7 per model on average, depending on the model complexity and the data. This GPU-based implementation of DCM for ERP gives qualitatively the same results as the original MATLAB implementation does at the group level analysis. In conclusion, we believe that the proposed GPU-based implementation is very useful for users as a fast screen tool to select the most likely model and may provide implementation guidance for possible future clinical applications such as online diagnosis.

## Introduction

Dynamic Causal Modelling for Event Related Potentials (DCM for ERP) [1], [2] is a recently developed advanced method embedded in SPM (Statistical Parametric Mapping; a MATLAB software package; http://www.fil.ion.ucl.ac.uk/spm) as a module for studying neuronal effective connectivity measured with EEG. DCM for ERP has been used to address the issues about the brain plasticity and functional asymmetries [3]–[5]. The basic idea of DCM for ERP is to fit the observed time series data with a spatiotemporal model, of which the temporal dynamics is formulated based on a neuronally plausible model, the Jansen model [6], and the neuronal network architecture is specified based on the prior knowledge of the user and the hypothesis been tested. The event related potentials are assumed as a result of the changes of the connection or coupling strength at each level of a cortical hierarchy in that spatiotemporal DCM model. This can be parameterized as a multiple-inputs multiple-outputs (MIMO) system, referred as the neuronal state equations in DCM for ERP. To solve these neuronal state equations, an iterative procedure named the expectation maximization (EM) algorithm [7], is employed to find the optimal model parameters that govern the underlying neuronal dynamics given a set of observed events (data) and the underlying probability model. The convergence will be reached when the likelihood function is maximized (for details, see [8]). Several models could be inverted according to the testing hypothesis, and Bayesian Model Comparison (BMS) allows one to select one winning plausible model that best explains the EEG data in terms of their model evidence. The computing workload is extremely heavy in DCM because of the compute-intensive EM algorithm and the possible combinatorial explosion of models to be tested [9]. Furthermore, while MATLAB could be efficient in numerical computations (for instance, matrices operations), it still has inherent limitation incurred by sequential execution. In fact, there are several attempts to parallelize the applications on MATLAB, such as image registration [10] and B-spline interpolation [11], and they have gained acceleration by a factor of about 4 to 13, depending on the applications.

Recently, the graphics processing units (GPUs) have been widely adopted as coprocessors to accelerate computation of CPUs, such as online biomedical applications [12], [13] and massive computing applications [13]–[20]. A GPU is an electronic device, which is initially developed for efficient manipulation of computer graphics. A GPU is notable for its multi-core architecture, and thus becomes a good tool to handle compute-intensive problems in parallel. A GPU contains several stream multi-processors (SMs) that can launch huge threads to process a computing task simultaneously in a single-instruction-multiple-data (SIMD) manner. The compute unified device architecture (CUDA) is a GPU programming model proposed by NVIDIA (CUDA C Programming Guide,” Available: http://developer.nvidia.com/nvidia-gpu-computing-documentation). In CUDA, the data-parallel portion of a program is executed as the kernel on the NVIDIA GPU device. The kernel is executed as a grid of thread blocks, and threads from different thread blocks cannot cooperate. A thread warp is the basic unit of execution in a block, which contains 32 threads in CUDA Toolkit 4.2 and the earlier versions. That is, an NVIDIA GPU can only run 32 threads simultaneously per block. As the amount of cores in a GPU is different, the number of GPU blocks which can work simultaneously is also different.

Since a GPU is a physically independent device to a CPU, the only way to communicate with each other is by transferring data among them. Before the GPU starts computation, the CPU, referred as the host, has to copy data and sends them to the GPU, referred as the device. The data transfer procedure is necessary and may incur performance hit that reduces the performance improvement from GPU parallelism. When the data arrives at the GPU, the data have to be stored in the GPU memory hierarchy. The NVIDIA GPU hierarchy consists of registers, local memory, global memory, and constant memory, and different types of memories have different features, of which the details are described in the NVIDIA CUDA programming Guide. If the user’s application does not specify the type of GPU memory for data storage, the GPU will save the data in the global memory on default. In the implementation of CUDA, a multiprocessor can execute multiple thread blocks in parallel; shared memory and registers are partitioned among the threads of all concurrent blocks accordingly. Since the shared memory and the registers are optimized for data access among a thread block, putting data in the shared memory and registers can reduce data access latency [21]. However, the size of the shared memory and the size of the registers are small. Only necessary and critical data should be put in the shared memory and the registers, while other data are put in the global memory or the memory in the host.

The goal of this study aims to use the GPU to reduce the execution time of DCM that is implemented in MATLAB. There are two kinds of approaches to do so. The first kind of approach is to use a high-level programming tool that supports direct GPU function invocations on MATLAB, such as JACKET (Jacket for GPU Computing – AccelerEyes. available at: http://www.accelereyes.com/products/jacket), parallel computing toolbox (PCT) (Parallel Computing Toolbox – Mathwork.” available at: http://www.mathworks.com/products/parallel-computing/), and GPUmat (available at: http://sourceforge.net/projects/gpumat/). The second kind of approach is to call external CUDA programs from MATLAB. The major advantage of the first approach is that there exist no barriers between GPU and MATLAB. However, this also puts some constraints and limitations on a developer’s code since one cannot design/optimize his own parallel strategy. On the contrast, the second approach allows a developer to design/optimize his own parallel strategy with better control on the GPU and the CPU. We have chosen the second approach in this study because it provides good flexibility and direct control on the GPC. We replaced part of the iterative EM algorithm, which is the most time consuming part of DCM for ERP, by invoking an external program written in CUDA and C++. In the implementation, we employed an adaptive computing thread allocation framework to fulfill a fast estimation of neural effective connectivity in DCM for ERP. To evaluate the performance efficacy of our proposed parallelism strategy, synthetic data with different model complexity and data length were generated. Ten sets of real experimental EEG data were used to test the computing accuracy and the speedup factor in practice. Both synthetic and experimental results showed that the proposed strategy can shorten the execution time.

## Materials and Methods

### DCM for ERP

In this study, we propose to accelerate DCM for ERP with a GPU using CUDA. In this section, we briefly describe the theory of DCM for ERP, in particular, the neuronal model and the EM algorithm, which is the parallel processing target. The neuronal model consists several pre-specified cortical sources and each cortical source comprises four cell populations – superficial and deep pyramidal cells, spiny stellate cells and inhibitory interneurons. These populations are coupled through the intrinsic connectivity among cortical layers and the hierarchical cortico-cortical network model of cortical sources can be constructed through three kinds of inter-area connections (forward, backward and lateral) according to the belief of the users about the data (i.e. the prior), the experimental manipulation and the hypothesis been tested. The changes of the connection or coupling strength at each level of a cortical hierarchy result in the event-related responses and this can be formulated by a series of differential equations [22]:
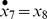


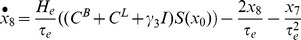


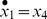


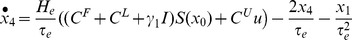


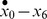


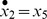


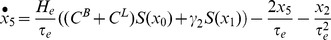


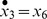


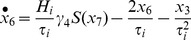
(1)


where each 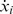 represents a state of neuronal subpopulations within one area, and the matrices *C^F^*, *C^B^* and C^L^ encode forward, backward and lateral extrinsic connections between areas, respectively. The deterministic inputs *u* corresponded to experimental manipulations (i.e., presentation of stimuli) were introduced to the state equations via input connections *C^U^* to perturb the system and subsequently evoke the neuronal responses. In addition, the experimental factors (i.e., stimulus attributes or context) can be encoded in *C^B^*.

The integration of the state equations gives the brain activities at the neuronal level and was transferred into the observation *h* through a modality-dependent biophysical transfer function *g* (for example, the leadfield matrix for EEG in DCM for ERP):
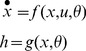
(2)


Inversion of this DCM for ERP model, given empirical data, makes possible the inferences about different models and the parameters of a particular model.

DCM utilizes an iterative procedure, the expectation maximization (EM) algorithm to search for the optimal parameters of the probability model in a heuristic manner, such that the likelihood function is maximized given a set of observed events (data) and the underlying probability model. The EM algorithm has two major steps, the expectation step (E-step) and the maximization step (M-step). The E-step calculates the current likelihood with the given/updated parameters; the M-step produces a new set of estimated parameters though maximizing the log-likelihood. The two steps continue in turn iteratively until convergence is reached, and then the EM algorithm outputs the searched optimal parameters (see [8] and [23] for details):

Until convergence {

E-step
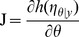


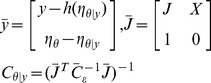


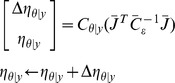



M -step
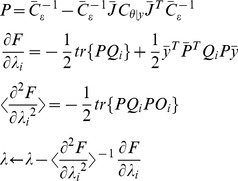
(3)where the explicit system Jacobian J in the E-step will then used to calculate the numerical solution of the neuronal state equations (see Equation 1) as shown in the following update scheme:

### Parallelization Strategy

**Figure 1:**
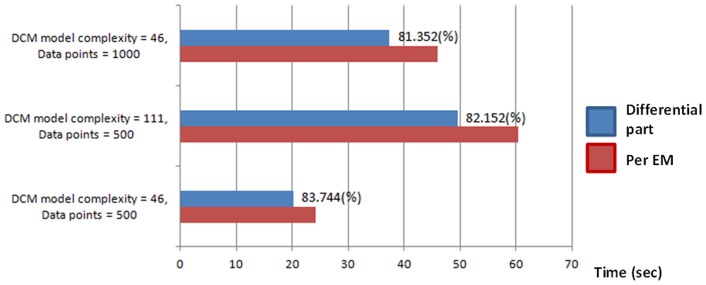
Evaluation of the most time-consuming portion in DCM for ERP (Per EM iteration) in Matlab.

**Figure 2:**
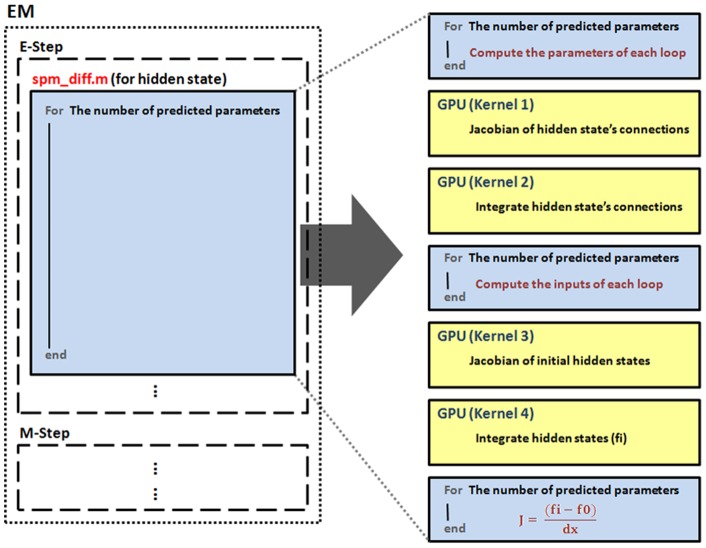
Schematic illustration of the parallelism strategy.

**Figure 3:**
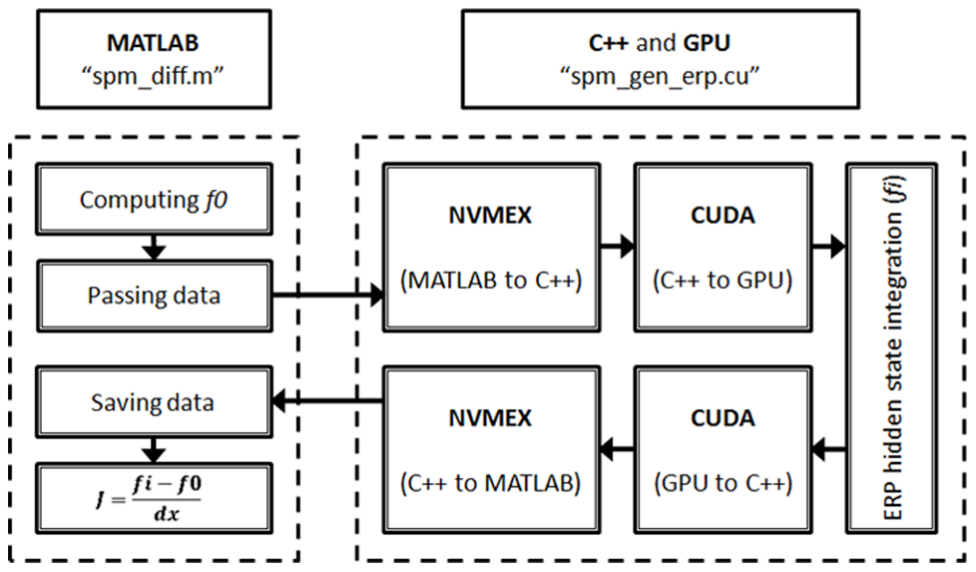
Schematic illustration of the data passing flow between the MATLAB and the GPU.

This study re-implemented part of DCM for ERP code using CUDA and improved the computing efficiency of DCM for ERP. As in DCM for ERP, the optimization scheme uses the MLE algorithm to iteratively update the model parameters and the computation inside each iterative loop of the MLE algorithm could be calculated in parallel [24]. Our strategy follows the above idea by first identifying the most time-consuming portion in the iterative loops of the EM algorithm and then parallelizing the calculation on the GPU. Specifically, the differential part that calculates the current estimates of the system Jacobin matrix J at neuronal source space in the E-step (see Equation 3) was selected to undergo the parallelism. This is because, by using synthetic data, our profiling records showed that this portion takes up to 80% of the total execution time in each EM loop in MATLAB, independent of data length and model complexity (i.e. the number of model parameters to be estimated) (figure 1). We further investigated this differential operation and found that, explicitly, the differential operation starts with the integration of the neuronal state equations given the current model parameters, followed by the mapping from neuronal states to cortical activities (Eq. 2) and then proceeds to the derivatives of the system Jacobian. It can be seen that the integration of the neuronal states is the best target for parallel computing given its usage and independency between states. Conceptually, we initiate the differential process in MATLAB, and then dispatch the computational tasks of state integrations to the GPU, along with the data for parallel processing. The result calculated by the GPU is then returned to MATLAB for subsequent calculation of the system Jacobian J. By the result, the EM algorithm continues to find a better set of parameters that maximizes the likelihood of the given model and the observed data. Figure 2 illustrates the scheme of the parallelism strategy. This strategy requires sending the data and assigning the computational tasks from the host (CPU) to the device (GPU). The communication between the host and the device is through NVIDIA NVMEX and the C++ code that executes the CUDA code (kernels) on the device. Figure 3 shows how the data are passed and computed between MATLAB and the GPU. To parallelize the E-Step for-loop, we first partition the for-loop into seven small for-loops, and then use CUDA to implement the four for-loops, Kernels 1∼4, which are suitable for parallel processing in the GPU. The remaining sequential code pieces in the E-Step are not parallelized in CUDA because they are very fast in MATLAB. We have observed that, parallelizing these code pieces usually causes negative impacts on the performance.

Note that the model to be inverted is specified by the users according to the hypothesis been tested, including the number of areas and the connection architectures. Therefore, the number of model parameters C^F^, C^B^ and C^L^, reflecting the model complexity denoted as N in Figure 2, cannot be determined beforehand. So, in this study, we have to employ an adaptive parallel strategy, which dynamically assigns the tasks to the thread blocks to boost up the computation performance. That is, we dynamically assign each GPU thread to handle one integration task of inferring neuronal states.

### Adaptive Hardware-Dependent Thread Arrangement

**Figure 4:**
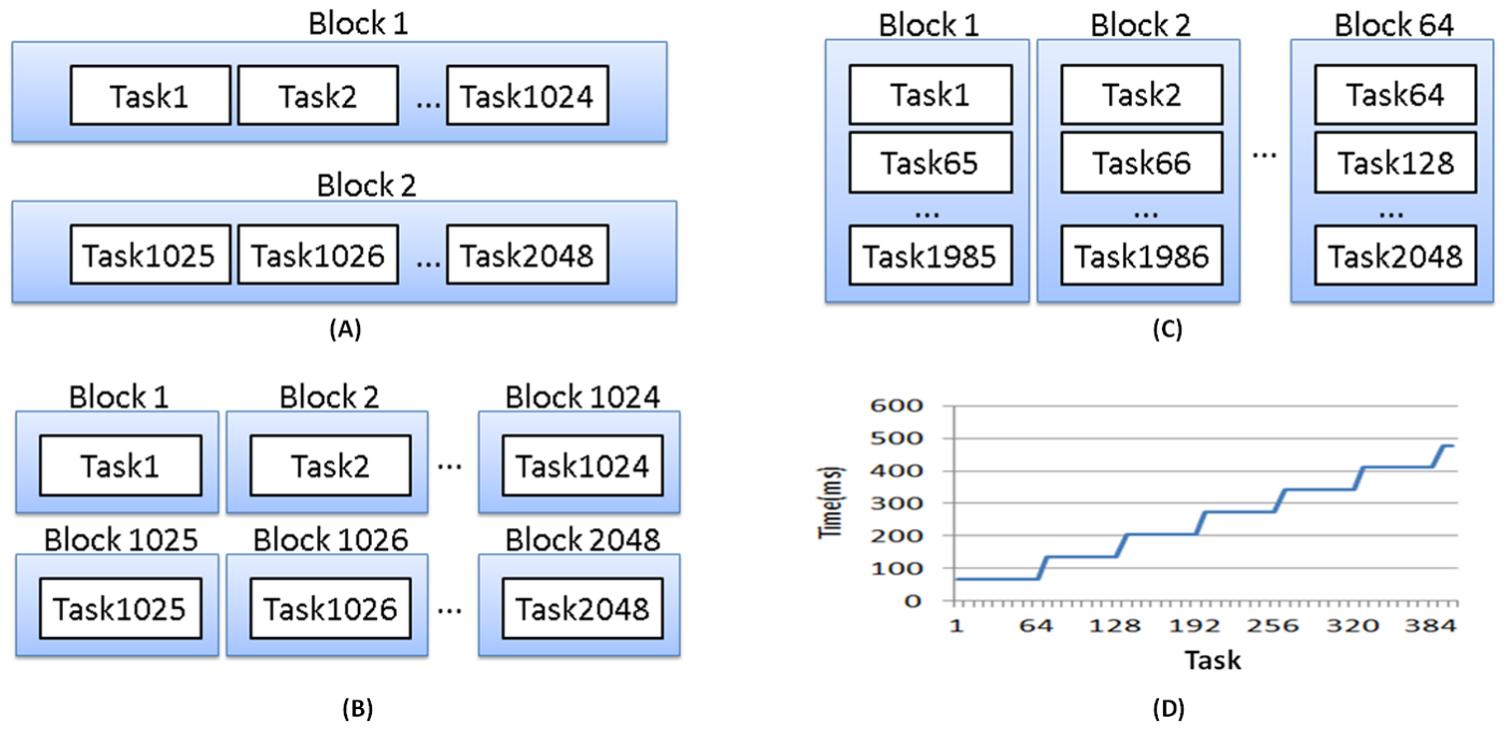
The architectures of three arrangements, (A) the block-first arrangement, (B) the thread-first arrangement, and (C) the block-64 arrangement, and the detection of the maximum number of simultaneous thread blocks using a small benchmark program (D).

In parallel computing, usually, each thread handles a computing task at a time. Without the knowledge of the underlying hardware, there are two naïve ways to arrange threads to computational tasks: (1) the thread-first arrangement strategy termed as thread and (2) the block-first arrangement termed as block. Figures 4(A) and 4(B) represents the concept of these two naïve arrangement strategies, respectively. Figure 4(A), the thread-first arrangement strategy, shows that two thread blocks are used to process 2048 independent tasks, where each thread block handles 1024 tasks. Under the thread-first arrangement, the threads are grouped as blocks, and a block must be fully occupied first and then the next block can be assigned to the remaining tasks. Because the maximum number of GPU threads per block is 1024 (or 512 for an older version of CUDA) and in this study the maximum number of tasks is seldom larger than 1024, we may use only one block by the thread-first arrangement strategy. Since a CUDA SM executes in the granularity of blocks, the thread-first strategy may not fully utilize the hardware resources of a GPU in this study. On the contrast, when employed the block-first arrangement strategy to process 2048 independent tasks, only the first thread of a block is used and each thread handles a task, as shown in Figure 4(B). Therefore, the number of blocks increases as the number of tasks increases.

The above two thread arrangement strategies are not efficient because they cannot utilize the hardware resources well. It has been observed that, the computation on the GPU becomes inefficient if the number of the blocks is not a multiple of the number of multiprocessors on the GPU [25]. Figure 4(D) shows the execution time of running a set of logarithm operations per thread block in the NVIDIA GTX 560 Ti GPU device, given different number of thread blocks. The execution time increases regularly as the number of thread blocks increases by a factor of 64. This means the maximum number of thread blocks that can be launched concurrently is 64. Since the maximum number of concurrent thread blocks is determined by the underlying GPU device, we can use a small program to detect the maximum number of concurrent thread blocks of the GPU device after the GPU device is installed in the computer. The detected value X is then used to assign threads to the computing tasks. The proposed thread arrangement strategy, termed as blockX, follows the concept of the block-first strategy, except that the number of blocks is limited to the detected value X. In the case that the number of tasks exceeds X, we use the round-robin approach to fairly assign threads to tasks until all of the X blocks reach the maximum number of threads per thread block, which is an inherent constraint in CUDA. When all the blocks are full of tasks, we will create another set of X thread blocks to handle the remaining tasks. Figure 4(C) shows the concept of the proposed blockX strategy for X = 64, and thus we named it the block64 strategy. As in typical DCM For ERP studies, the number of parameters are usually over 64 but less than 1024, the proposed hardware-dependent thread arrangement strategy (the blockX strategy) is able to achieve good load balance among all the major computing components, the streaming multiprocessors. Therefore, the blockX strategy theoretically achieves better performance than the two naïve thread arrangement strategies.

Note that the value X can be calculated from the hardware specification of a GPU card. The maximum number of resident (simultaneous) thread blocks in a Streaming Multiprocessor is 8 for the GPU cards with CUDA computing capability 1.x or 2.x; the maximum number of resident thread blocks in a Streaming Multiprocessor is 16 for the GPU cards of CUDA computing capability 3.x. The GPU card used in our simulations complies with the CUDA computing capability 2.x, and the number of Streaming Multiprocessors on the card is 8. Therefore, the maximum number of resident (simultaneous) thread blocks is 8×8 = 64. As many users of the program DCM for ERP may not care to know the hardware specification of a GPU card, it would be more user-friendly and efficient to decide the maximum number of simultaneous thread blocks automatically by using a small benchmark program to detect it.

### Simulation data

The data used in the simulation was downloaded from SPM website (http://www.fil.ion.ucl.ac.uk/spm/), in which 128 channel electroencephalography (EEG) signal was measured during an auditory oddball paradigm for eliciting Mismatch Negativity activities (see [3] for details). The model used for evaluating the execution time has five areas and reciprocal connections between low and higher areas and the task-specific modulations were set to be forward and backward (as FB model in [3]). This setting results in 46 model parameters to be estimated as shown in Figure 1. To increase the model complexity, we simply allow all possible connections and get the model complexity of 111 under this configuration (Figure 1). For more complex model (i.e. model parameters >111), we manually force the number of parameters to match our design. To increase the data length, we just concatenate the same epoch several times and truncate it to fit the desired data length. Note that, in these simulations, our design was only for evaluation of the execution time but not the accuracy.

### Empirical data

**Figure 5:**
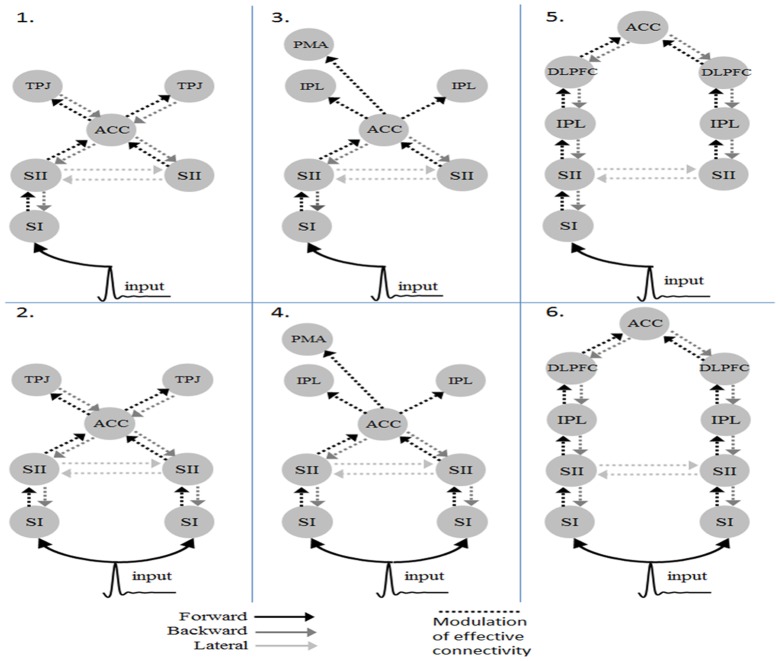
The models used in the performance evaluation (SI: primary sensory area; SII: secondary sensor area; ACC: Anterior cingulate cortex; TPJ: Temporoparietal junction; IPL: Inferior parietal lobule; PMA: Premotor area; DLPFC: Dorsolateral prefrontal cortex).

The real experimental data were used to test the computing accuracy and the speedup factor in practice. The 32 channel EEG data were measured with 250 Hz sampling rate during a 3-D Virtual Reality based ball catch task. Ten healthy right-handed volunteer subjects participated in this experiment and part of the data will be published for addressing other scientific issues later. All subjects gave written informed consent for the experiment with a protocol approved by the institutional review board of the Taipei Veterans General Hospital. They were instructed to perform a ball catch task using their dominant hand under the virtual reality environment. The ball catch task has two conditions: standard and rare, which differ in their occurrence frequency. In the standard condition (80% of occurrence), the subjects have to catch a ball and when it is successful, the subjects will receive a sensational force through the hepatic device. For 20% of total events, there will have no force feedback even the subjects have caught the ball successfully. This oddball paradigm is to elicit a sensory P300 cortical activities. The data were epoched offline, with a peristimulus window of 0 to 900 ms, filtered with 30 Hz low-pass filter, artefact removal using the fully automated correction method [26] and averaged across artefact-free trials. For DCM for ERP analysis, we first specify six plausible models (Figure 5), differed in the areas and the inputs based on three previous literatures [27]–[29], to identify the most likely model hierarchy. The experimental data were used to evaluate the speedup factor and the estimation accuracy in practice in terms of the model evidence by Bayesian Model Selection (BMS).

## Results

In this study, we proposed a computing scheme using external calls from MATLAB to the GPU to achieve a fast estimation of neural effective connectivity in DCM for ERP. Synthetic data were used to evaluate the computing efficacy of the GPU in terms of the speedup factor and the experimental data were used to validate the accuracy of the computation in the GPU and quantified the speedup factor in practice.

### Simulation result

#### Impact of thread arrangements on execution time

**Figure 6:**
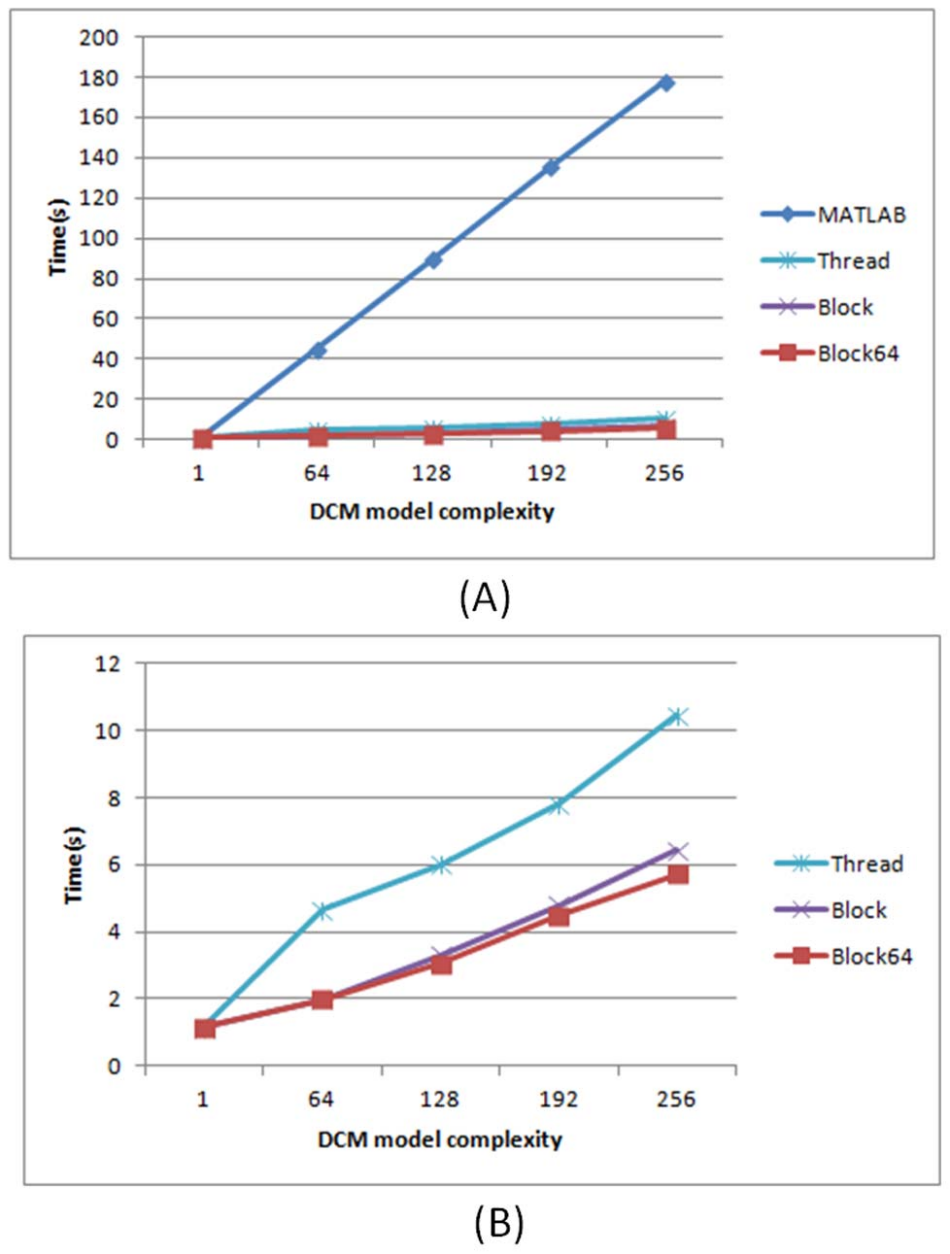
The GPU computing efficiency of the three different thread arrangements in comparison with the original MATLAB implementation.

**Figure 7:**
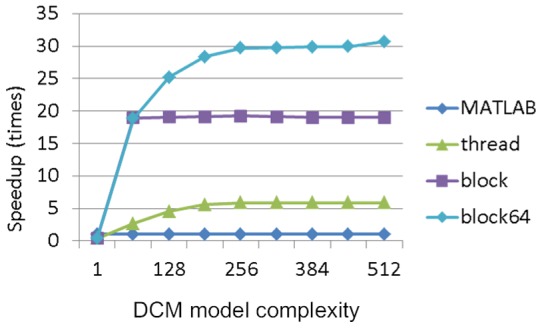
The effect of the number of tasks (model complexity) in relation to speedup factor.

We used the NVIDIA GTX 560 Ti GPU device to analyse the synthetic data for DCM for ERP in parallel. The NVIDIA GTX 560 Ti GPU device has 8 streaming multiprocessors and allows at most 8 resident thread blocks per streaming multiprocessor. Based on the result of our detection program, we found that *X* = 64 achieves better performance for the proposed blockX thread arrangement strategy. The relationship between the number of tasks (complexities) and the performance of the proposed parallel computing strategies was first evaluated in terms of execution time per spm_diff function call (Figure 2) using synthetic data. Figure 6 shows the GPU efficiency resulting from the three thread arrangement strategies in comparison with the original MATLAB implementation, given 500 points of time series data. It can be seen that, with the external function calls to the GPU, the execution time reduces dramatically for all the three thread arrangement strategies (Figure 6(A)). We then further examine the superiority of the proposed blockX (block64 in this case) strategy in execution time while comparing it to the block-first strategy in Figure 6(B). The best speedup factor of them occurs when the number of tasks is a multiple of 64, and the block64 strategy performs better when the number of tasks exceeds 64. This is the evidence that the hardware constraints affect the performance of the software implementation. The speedup factors of the three arrangements are shown in Figure 7. The proposed hardware-dependent thread arrangement strategy (block64) can accelerate the computation by up to a factor of 31, depending on the model complexity.

**Figure 8:**
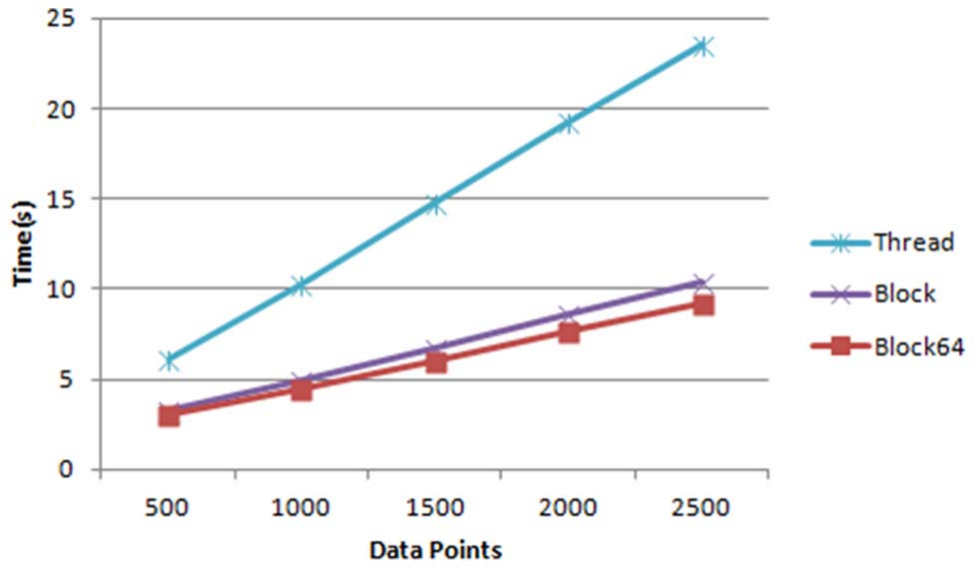
The effect of data length in relation to the execution time.

**Figure 9:**
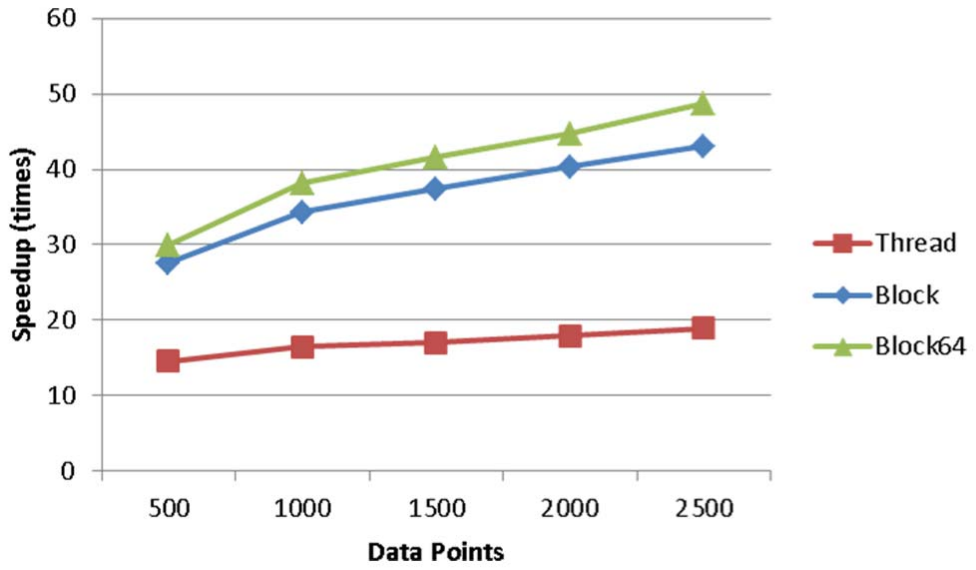
The effect of data length in relation to speedup factor.

**Figure 10:**
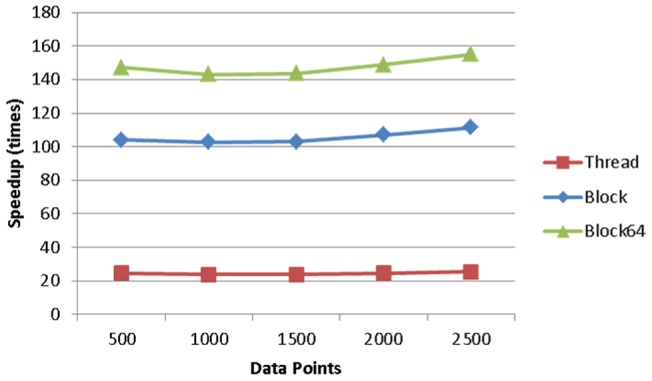
The effect of data length in relation to speedup factor for the parallelized part of DCM for ERP.

The effect of data length in relation to the execution time is illustrated in Figure 8, given the model complexity of 128. The execution time increases linearly with the data length, and the block64 strategy has the slowest increasing slope, implying it has the best performance. This is confirmed by Figure 9, where the block64 strategy outperforms the others and reaches about 49 times of speedup based on the execution time of the MATLAB implementation. When we only compare the speedup of the code pieces that are parallelized by different thread arrangement strategies, as shown in Figure 10, the block64 strategy outperforms the others by a large margin and reaches about 155 times of speedup.

**Figure 11:**
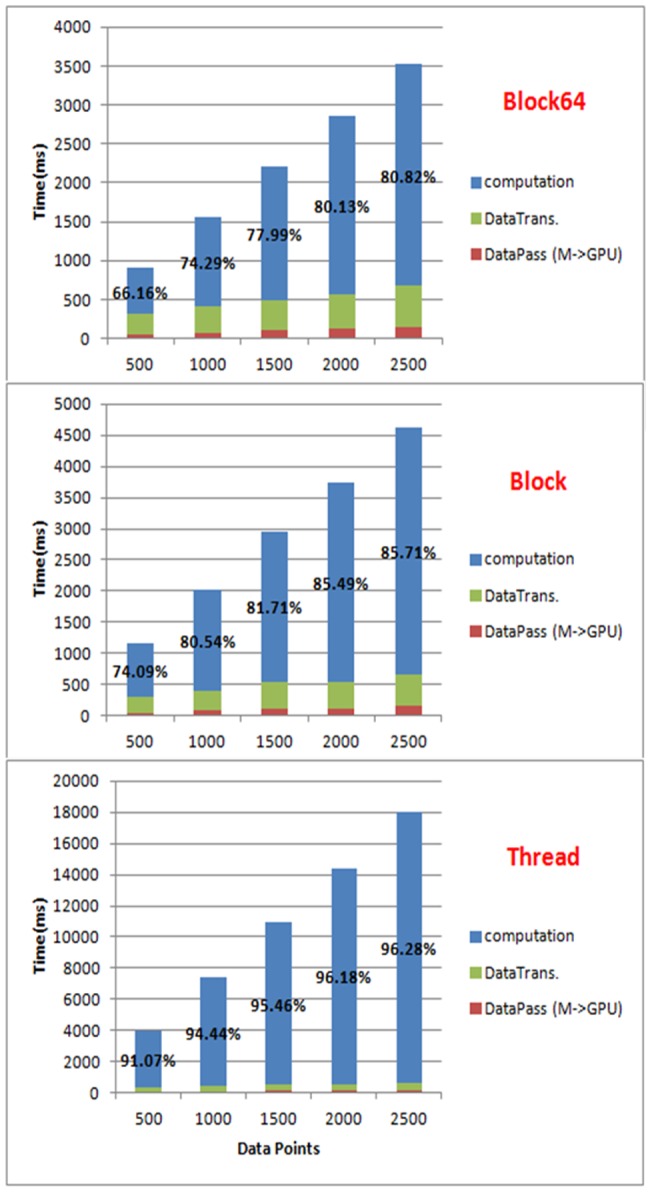
Time span composition, including computing time (in blue), data transformation (in green) and data passing (in red), of the three arrangements.

We further investigated the time span composition of the three thread arrangement strategies to reassure the efficiency of the block64 strategy. Figure 11 illustrates the breakdown of the execution time in different phases: computing, data transformation (from matrices to vectors for GPU computing) and data passing (between MATLAB and GPU). As the time for data transformation and data passing are the same for all the three thread arrangement strategies, the computing phase of block64 takes only up to 80% of the total execution time while the other two could use up 90 of it, though it relates to data length.

### Experimental results

#### Computational accuracy and efficiency

**Figure 12:**
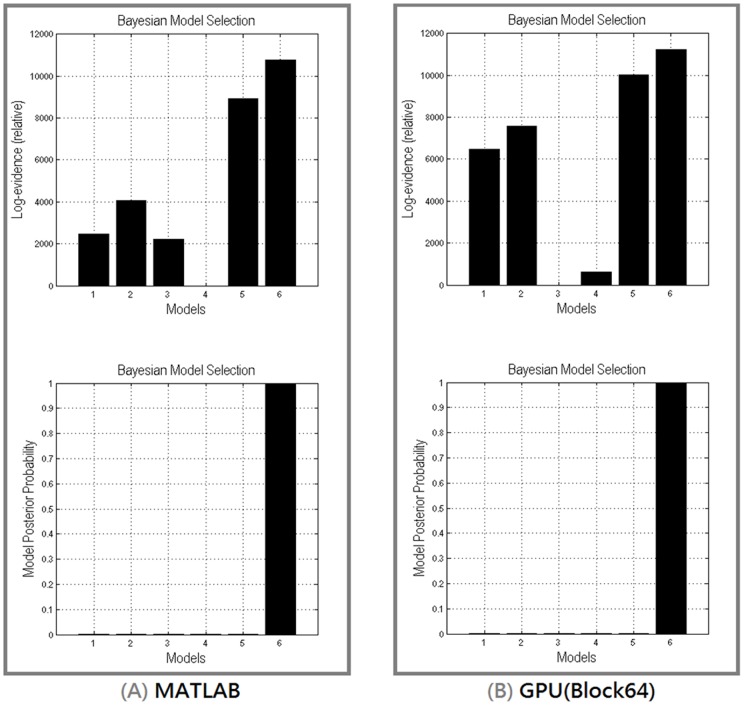
The group results of BMS under fixed effect assumption from MATLAB (A) and GPU (B).

Six DCM models were inverted using the MATLAB implementation and the CUDA-GPU (block64) implementation, for each subject as described in the previous section (see Figure 5). At the single subject level, six out of ten subjects have the model 6 as the best model from the results of the MATLAB implementation and only 4 subjects have Model 6 as the winning model from the results of the CUDA-GPU implementation. At the group level, the summed log-evidences over 10 subjects from the MATAB and the CUDA-GPU implementations are shown in Figure 12 (up panel). Bayesian model selection demonstrated that both, under fixed effect assumption, have chosen the model 6 (P > 0.99) as the best model, followed by model 5 and then model 2 (Figure 7, lower panel). When comparing the model-evidence of the same data and the same model between the MATLAB implementation and the CUDA-GPU implementation, the MATLAB implementation has 33 out of 60 (10 subjects * 6 models) greater values while the CUDA-GPU implementation has 27. Two sample t-test confirmed that, there has no significant difference (t = 0.4362, p = 0.66) on the model evidence between the results of both implementations. In other words, qualitatively, the CUDA-GPU implementation gives the same results as the MATLAB implementation does. We then compared the coupling parameters estimated by both implementations and found that, given the same initial priors, they didn't converge to the same results (Table 1). This may be due to the fact that, the two implementations have different numerical accuracy and lead to be trapped in different local maximum.

**Table 1:** One example of the different estimates from MATLAB and GPU.

Forward			Backward		
Connection	MATLAB (A. U.)	GPU (A.U.)	Connection	MATLAB (A. U.)	GPU (A.U.)
LS1 -> LS2	−0.357	−0.593	LS2 -> LS1	0.099	0.059
RS1 -> RS2	−0.366	−0.289	RS2 -> RS1	−0.023	0.134
LS2 ->LIPL	−0.123	−0.299	LIPL -> LS2	−0.091	0.081
RS2 -> RIPL	−0.461	−0.317	RIPL -> RS2	−0.194	0.024
LIPL -> LDLPFC	−0.135	0.228	LDLPFC -> LIPL	−0.517	−0.062
RIPL -> RDLPFC	−0.149	−0.411	RDLPFC -> RIPL	−0.123	−0.006
LDLPFC -> ACC	−0.111	0.520	ACC -> LDLPFC	−0.046	−0.148
RDLPFC -> ACC	−0.469	0.314	ACC -> RDLPFC	−0.031	0.127

Lateral			Input		
Connection	MATLAB (A. U.)	GPU (A.U.)	Connection	MATLAB (A. U.)	GPU (A.U.)
RS2 -> LS2	−0.009	0.064	Input -> LS1	−0.505	−0.541
LS2 -> RS2	−0.008	−0.185	Input -> RS1	−0.859	−0.329

In relation to the reduced execution time, the GPU can shorten the total execution time by a factor of 7 per model or 6 per EM loop on average. Table 2 shows the detailed information of the speedup factors of the GPU block64 strategy for each model. Note that the time saved by the GPU is data- and model- dependent since the EM algorithm may execute different number of loops for a different case.

**Table 2:** The speedup factor of the GPU block64 strategy for each model (of one representative subject).

Model No.	Number of EM loops per model		Execution time per EM loop			Total execution time per model		
	MATLAB (loops)	GPU (loops)	MATLAB (s)	GPU (s)	Speedup^a^ (times)	MATLAB (s)	GPU (s)	Speedup^b^ (times)
1	21	60	90.97	9.80	9.28	1910.30	588.28	3.24
2	15	49	85.56	12.18	7.02	4623.80	451.73	10.24
3	13	37	63.68	9.21	6.91	827.82	340.68	2.43
4	52	27	91.76	22.42	4.09	4771.70	605.31	7.88
5	48	28	96.33	16.13	5.97	4623.80	451.73	10.24
6	32	51	120.06	23.39	5.13	6123.10	748.37	8.18
Avg.	30.17	42.00	91.39	15.52	6.40	3813.42	531.02	7.04

speedup^a^: speedup factor per EM loop ; speedup^b^: speedup factor of total execution time.

## Discussions

In this study, we proposed a computing scheme using external calls from MATLAB to the GPU to achieve a fast estimation of neural effective connectivity in DCM for ERP. Synthetic data were used to evaluate the computing efficacy of the GPU in terms of the speedup factor. The simulations show that this proposed GPU-based parallel computing strategy can accelerate the computation by a factor of about 155 for the parallel part and about 49 per EM loop. The impact of data length on the execution time in terms of speedup factor is minor, in the fact that the execution time increases linearly with the data length. The experimental data were then used to validate the accuracy of the computation in the GPU and quantified the speedup factor in practice. The speedup factor using real experimental data achieves about 7 per model, depending on the model complexity and the data length. This GPU-based implementation of DCM for ERP gives qualitatively the same results as MATLAB does in model selection though quantitatively the estimates of model parameters are not equal.

### GPU computing accuracy

Qualitatively, the GPU-based DCM for ERP gives the same results at the group level analysis as what MATLAB does. However, at the single subject level, the estimates of model parameters vary considerably. The reason for the difference between the MATLAB implementation and the GPU implementation, shown in Table 1, is due to the fact that the intrinsic numerical accuracy is different between MATLAB and the GPU, despite they both use double precision floating-point for computation. The difference is trivial in the beginning (<10^−5^) but is further amplified by the iterative procedure in the EM algorithm. Furthermore, as in DCM for ERP, the Restricted maximum likelihood (ReML) approach was used in the optimization for degrading the importance of the nuisance parameters and this makes the likelihood function in DCM for ERP sensitive to the change of model parameters, i.e. the system Jacobian. Both together result in different gradient direction and convergence paths in MATLAB and the GPU as shown in the iteration number in EM in Table 2, for instance, 21 iterative loops in MATLAB and 60 iterative loops in the GPU for Model 1. Nevertheless, the accuracy of GPU computing is fair as there has no significant difference of model evidence between them at the group level.

### GPU speedup factor

The computing efficiency of our GPU implementation in terms of execution time is much better than the original MATLAB implementation of DCM for ERP, and the performance of the proposed blockX thread arrangement strategy is the best among the three thread arrangement strategies mentioned in the previous sections. The speedup factor varies significantly with a range from a few hundred in the parallel part using synthetic data down to about 7 per one plausible DCM model in real EEG data. The notable decrease of computing performance in practice using experimental data is because there are still many parts which can't be parallelized (or is not worthy) in the computing of DCM for ERP. These parts still take up a lot of computing time, for example, the matrix calculation, even though this part only takes less than 20% of the execution time in the original MATLAB implementation. As MATLAB has be known for its ability to handle complex matrix operations efficiently, we simply take this advantage by leaving the complex matrix operation in MATLAB to boost the most computing gain of both, as seen in Figure 2. This results in the best overall execution time per model. In addition, the different convergent paths also add the variability to the GPU speedup factor in practice. From the simulation, it can be inferred that the impact of data length on reducing execution time is minor in real data applications, as the typical EEG epoch is usually less than 2500 points. Nevertheless, the more complex the model is, the better the performance of the proposed blockX (block64 in all our simulation and experiment) strategy will achieve until all GPU resources are fully utilized.

Apart from the implementation consideration, the other important factor that affects the GPU performance is the time used for data transmission. We have observed that data transformation and data passing could be a performance bottleneck of our GPU implementations. It consumes a lot of time to pack and send data from the CPU to the GPU and this may reduce the benefits of GPU parallelism, especially when the data length is short. Furthermore, if the data type of the transmitted data is double-precision, the transmission speed can be 8 times as slow as that of single-precision data based on our measures. Because our goal is to maintain the same computing precision in the GPU as in MATLAB, our GPU implementation must suffer slow transmission speed to transmit double-precision data between the CPU and the GPU. In addition, the CUDA C programming guide also indicates that an NVIDIA GPU works much more efficient on single-precision data than on double-precision data. This means our GPU implementation can achieve better computing efficiency if we adopt single-precision data instead of double-precision data.

### GPU memory hierarchy and data layout

In the CUDA architecture, the data to be processed are sent from the main memory of the CPU to the global memory of the GPU. Each thread block has its shared memory, and all threads in a block can access the shared memory. Accessing the shared memory is much faster than accessing the global memory. However, to access the shared memory, one has to copy the data from the global memory to the shared memory. This procedure may incur overheads from data transmission and shared memory management. A GUDA program can be benefited from putting data in the shared memory if the data are frequently used and aligned well to reduce "bank conflicts" (a bandwidth problem caused by the hardware limitation) of the shared memory. Unfortunately, it is hard to use the GPU memory hierarchy to improve the efficiency of our parallel version of DCM of ERP. We have identified two difficulties in this problem domain. First, the data to be processed on the GPU by our DCM for ERP implementation consist of several data arrays of different sizes. The data are retrieved from the MATLAB computing environment and then sent to the GPU device. To avoid possible "bank conflicts" in the shared memory, the data have to be re-organized. However, data re-organization takes time, which may not be covered by the benefit of using the shared memory. Moreover, the data that are sent to GPU for parallel processing are not used frequently enough to reduce execution time. Importantly, the global memory becomes more efficient since CUDA computing capability 2.x. This may help to explain why our attempt of using shared memory does not work well. Nevertheless, in general, it is possible to use the shared memory to improve the execution time by putting some of the most frequent data items in the shared memory, though the cost of re-engineering is high and the gain is small in DCM for ERP.

## Conclusion

In conclusion, we have proposed a computing scheme using external calls to the GPU to achieve a fast estimation of neural effective connectivity in DCM for ERP. The speedup factor of GPU computing varies significantly with a range from 155 in the parallel part using synthetic data, and down to 7 per one plausible DCM model in real EEG data, depending heavily on the model complexity and the data length. This GPU-based DCM for ERP implementation gives qualitatively the same results as MATLAB does in model selection though quantitatively the estimates of model parameters are not equal. As the proposed hardware-dependent thread arrangement strategy could yield the computing efficiency of DCM for ERP while maintaining the accuracy and fidelity, we consider this a fast screen tool for users to select the most likely model and may provide implementation guidance for possible future clinical applications such as online diagnosis.

## References

[1] David O, Kiebel SJ, Harrison LM, Mattout J, Kilner JM, et al (2006) Dynamic causal modeling of evoked responses in EEG and MEG. Neuroimage 30: 1255–1272. 16473023 10.1016/j.neuroimage.2005.10.045

[2] Kiebel SJ, Garrido MI, Moran R, Chen CC, Friston KJ (2009) Dynamic causal modeling for EEG and MEG. Hum Brain Mapp 30: 1866–1876. 19360734 10.1002/hbm.20775 PMC6870752

[3] Garrido MI, Kilner JM, Kiebel SJ, Friston KJ (2007) Evoked brain responses are generated by feedback loops. Proc Natl Acad Sci U S A 104: 20961–20966. 18087046 10.1073/pnas.0706274105 PMC2409249

[4] Garrido MI, Kilner JM, Kiebel SJ, Stephan KE, Baldeweg T, et al (2009) Repetition suppression and plasticity in the human brain. Neuroimage 48: 269–279. 19540921 10.1016/j.neuroimage.2009.06.034 PMC2821573

[5] Brown HR, Friston KJ (2012) Dynamic causal modelling of precision and synaptic gain in visual perception – an EEG study. Neuroimage 63: 223–231. 22750569 10.1016/j.neuroimage.2012.06.044 PMC3438451

[6] Jansen BH, Rit VG (1995) Electroencephalogram and visual evoked potential generation in a mathematical model of coupled cortical columns. Biol Cybern 73: 357–366. 7578475 10.1007/BF00199471

[7] Moon T, Stirling W (1999) Mathematical Methods and Algorithms for Signal Processing: Prentice Hall.

[8] Friston KJ, Harrison L, Penny W (2003) Dynamic causal modelling. Neuroimage 19: 1273–1302. 12948688 10.1016/s1053-8119(03)00202-7

[9] Lohmann G, Erfurth K, Muller K, Turner R (2012) Critical comments on dynamic causal modelling. Neuroimage 59: 2322–2329. 22001162 10.1016/j.neuroimage.2011.09.025

[10] Huang TY, Tang YW, Ju SY (2011) Accelerating image registration of MRI by GPU-based parallel computation. Magn Reson Imaging 29: 712–716. 21531103 10.1016/j.mri.2011.02.027

[11] Dasgupta A, Kim H, Rorden C (2010) SPM & fMRI Medical Image Processing – GPU Computing Examples.

[12] Men C, Gu X, Choi D, Majumdar A, Zheng Z, et al (2009) GPU-based ultrafast IMRT plan optimization. Phys Med Biol 54: 6565–6573. 19826201 10.1088/0031-9155/54/21/008

[13] Wilson JA, Williams JC (2009) Massively Parallel Signal Processing using the Graphics Processing Unit for Real-Time Brain-Computer Interface Feature Extraction. Front Neuroeng 2: 11. 19636394 10.3389/neuro.16.011.2009 PMC2715290

[14] Kim D, Trzasko JD, Smelyanskiy M, Haider CR, Manduca A, et al (2010) High-performance 3D compressive sensing MRI reconstruction. Conf Proc IEEE Eng Med Biol Soc 2010: 3321–3324. 21096822 10.1109/IEMBS.2010.5627493

[15] Mousazadeh H, Marami B, Sirouspour S, Patriciu A (2011) GPU implementation of a deformable 3D image registration algorithm. Conf Proc IEEE Eng Med Biol Soc 2011: 4897–4900. 22255436 10.1109/IEMBS.2011.6091213

[16] Raimondo F, Kamienkowski JE, Sigman M, Fernandez Slezak D (2012) CUDAICA: GPU optimization of Infomax-ICA EEG analysis. Comput Intell Neurosci: 206972. 10.1155/2012/206972 PMC3395116 22811699

[17] Yang J, Feng C, Zhao D (2013) A CUDA-based reverse gridding algorithm for MR reconstruction. Magn Reson Imaging 31(2): 313–323. 22898698 10.1016/j.mri.2012.06.038

[18] Zhuge Y, Cao Y, Udupa JK, Miller RW (2011) Parallel fuzzy connected image segmentation on GPU. Med Phys 38: 4365–4371. 21859037 10.1118/1.3599725 PMC3188606

[19] Dudley JT, Butte AJ (2009) A quick guide for developing effective bioinformatics programming skills. PLoS Comput Biol 5: e1000589. 20041221 10.1371/journal.pcbi.1000589 PMC2791169

[20] Pinto N, Doukhan D, DiCarlo JJ, Cox DD (2009) A high-throughput screening approach to discovering good forms of biologically inspired visual representation. PLoS Comput Biol 5: e1000579. 19956750 10.1371/journal.pcbi.1000579 PMC2775908

[21] Nunna S, Bordoloi UD, Chakraborty S, Eles P, Peng Z (2010) Exploiting GPU On-Chip Shared Memory for Accelerating Schedulability Analysis. 2010: 147–152.

[22] Bastos A, Moran R, Litvak V, Fries P, Friston KJ (2011) A Dynamic Causal Model of how inter-areal synchronization is achieved in canonical microcircuits.; Society for Neuroscience 2011.

[23] Friston KJ (2002) Bayesian estimation of dynamical systems: an application to fMRI. Neuroimage 16: 513–530. 12030834 10.1006/nimg.2001.1044

[24] Wang WJ, Chang YS, Wu CH, Kang WX (2012) A Self-Adaptive Computing Framework for Parallel Maximum Likelihood Evaluation. Journal of Supercomputing 61: 67–83.

[25] Preis T, Virnau P, Paul W, Schneider J (2009) GPU accelerated Monte Carlo simulation of the 2D and 3D Ising model,. Journal of Computational Physics 228: 4468–4477.

[26] Schlogl A, Keinrath C, Zimmermann D, Scherer R, Leeb R, et al (2007) A fully automated correction method of EOG artifacts in EEG recordings. Clin Neurophysiol 118: 98–104. 17088100 10.1016/j.clinph.2006.09.003

[27] Crottaz-Herbette S, Menon V (2006) Where and when the anterior cingulate cortex modulates attentional response: combined fMRI and ERP evidence. J Cogn Neurosci 18: 766–780. 16768376 10.1162/jocn.2006.18.5.766

[28] Downar J, Crawley AP, Mikulis DJ, Davis KD (2000) A multimodal cortical network for the detection of changes in the sensory environment. Nat Neurosci 3: 277–283. 10700261 10.1038/72991

[29] Huang MX, Lee RR, Miller GA, Thoma RJ, Hanlon FM, et al (2005) A parietal-frontal network studied by somatosensory oddball MEG responses, and its cross-modal consistency. Neuroimage 28: 99–114. 15979344 10.1016/j.neuroimage.2005.05.036

